# Dietary Creatine Supplementation in Gilthead Seabream (*Sparus aurata*) Increases Dorsal Muscle Area and the Expression of *myod1* and *capn1* Genes

**DOI:** 10.3389/fendo.2019.00161

**Published:** 2019-03-28

**Authors:** Lourenço Ramos-Pinto, Graciliana Lopes, Vera Sousa, L. Filipe C. Castro, Denise Schrama, Pedro Rodrigues, Luísa M. P. Valente

**Affiliations:** ^1^ICBAS-UP, Instituto de Ciências Biomédicas de Abel Salazar, Universidade do Porto, Porto, Portugal; ^2^Centro Interdisciplinar de Investigação Marinha e Ambiental/CIMAR, Interdisciplinary Centre of Marine and Environmental Research, Novo Edifício do Terminal de Cruzeiros do Porto de Leixões, Matosinhos, Portugal; ^3^Department of Biology, Faculty of Sciences (FCUP), University of Porto, Porto, Portugal; ^4^Centre of Marine Sciences of Algarve (CCMAR), University of Algarve, de Gambelas, Faro, Portugal; ^5^Department of Chemistry and Pharmacy, University of Algarve, de Gambelas, Faro, Portugal

**Keywords:** calpains, creatine supplementation, muscle growth and differentiation, myogenesis, myogenic differentiation 1 (*myod1*), myogenic regulatory factors (MRFs)

## Abstract

Creatine (Cr) is an amino acid derivative with an important role in the cell as energy buffer that has been largely used as dietary supplement to increase muscle strength and lean body mass in healthy individuals and athletes. However, studies in fish are scarce. The aim of this work is to determine whether dietary Cr supplementation affects muscle growth in gilthead seabream (*Sparus aurata*) juveniles. Fish were fed *ad libitum* for 69 days with diets containing three increasing levels of creatine monohydrate (2, 5, and 8%) that were compared with a non-supplemented control (CTRL) diet. At the end of the trial, the fast-twist skeletal muscle growth dynamics (muscle cellularity) and the expression of muscle-related genes were evaluated. There was a general trend for Cr-fed fish to be larger and longer than those fed the CTRL, but no significant differences in daily growth index (DGI) were registered among dietary treatments. The dorsal cross-sectional muscle area (DMA) of fish fed Cr 5 and Cr 8% was significantly larger than that of fish fed CTRL. The groups supplemented with Cr systematically had a higher relative number of both small-sized (≤20 μm) and large-sized fibers (≥120 μm). Dorsal total fibers number was highest in fish fed 5% Cr. In fish supplemented with 5% Cr, the relative expression of myogenic differentiation 1 (*myod1*) increased almost four times compared to those fed the CTRL diet. The relative expression of calpain 3 (*capn3*) was highest in fish fed diets with 2% Cr supplementation, but did not differ significantly from those fed the CTRL or Cr 5%. The *myod1* gene expression had a positive and significant correlation with that of *capn1, capns1a*, and *capn3* expression. These results suggest that the observed modulation of gene expression was not enough to produce a significant alteration in muscle phenotype under the tested conditions, as a non-significant increase in muscle fiber diameter and higher total number of fiber was observed, but still resulted in increased DMA. Additional studies may be required in order to better clarify the effect of dietary Cr supplementation in fish, possibly in conjunction with induced resistance training.

## Introduction

In the last two decades, the amount of captured fish has stagnated, whereas fish produced in aquaculture has been increasing (1). Several seabream species are farmed worldwide due to their savory meat and to meet its growing consumption trend. Among Sparidae, the gilthead seabream (*Sparus aurata*, L.) is one of the most important farmed fish species in the Mediterranean region with an estimated production of 160.563 tons in 2016 (2, 3).

Skeletal muscle represents 40–60% of the fish body mass and represents the edible part of the fish (filet). High growth performance and flesh quality are crucial for the success of the aquaculture industry. It is known that consumers show a preference for fresh fish with a firm texture (4). Several studies have reported the relationship between the muscle fiber size and the firmness of the flesh (5–7). In Atlantic salmon, Johnston et al. (8) demonstrated that the firmness and the color of a smoked filet were positively correlated with the muscle fiber density. Likewise, in gilthead seabream, flesh firmness positively correlated with both the fiber density and the number of small fiber but showed a negative correlation with skeletal muscle diameter (9). Nutrient availability is one of the most important factors influencing the muscle growth performance in fish. Therefore, the need to establish the most favorable rearing conditions, to produce robust fish that grow fast and have a texture able to fulfill consumer's expectations, is of major importance for the farming industry.

Creatine (Cr) is an amino acid derivative naturally synthesized in vertebrates from methionine, glycine, and arginine (10). It combines with inorganic phosphate to form phosphocreatine (PCr), which is mainly stored in skeletal muscle (~95%) (11, 12). Importantly, Cr is a physiological compound and is a part of the ATP/PCr phosphate energy system. PCr is a donor of phosphate to ADP for energy production and is controlled by creatine kinase (CK) that catalyzes the reversible reaction of the energy transfer pathway known as the CK/PCr energy shuttle, which provides immediate replenishment of ATP via high-energy phosphate compounds (13). Since skeletal musculature is a high-energy demand tissue, Cr plays an important role in muscle fibers as an energy buffer and also acts indirectly on muscle growth and strength by increasing the energy availability.

In humans, Cr analogs have proved to display important biological activities acting synergistically with some pharmaceutical formulations available in the market (11). In addition, it is well known that the oral ingestion of Cr-rich items, such as meat and fish, or via dietary supplements, will increase the whole body Cr pool (14). Studies have shown that Cr ingestion in humans can significantly increase the amount of physical work that can be performed, and hence, the athletes use Cr as a performance-boosting supplement (11, 12, 14). Currently, Cr supplementation in humans, in conjunction with heavy training exercise, was found to increase type I and II muscle fiber area, satellite cell number, myonuclei concentration, and type I and II myosin heavy chain (mhc) mRNA transcripts and protein content (15–18). Recent studies have also found that when subjects boost their muscle Cr levels via supplementation, they also increase the secretion of growth hormone (gh) and the expression of IGF-I at rest with no additional effect of exercise (19, 20). In fish, the effects of Cr on muscle growth have been poorly evaluated, but gh plays an important role in protein synthesis via the interaction with the growth hormone receptor (*ghr*) on the cell membrane (21), which are regulated during starvation and refeeding of rainbow trout (22). Gh induces muscle growth by modulating the expression of several genes belonging to the myostatin (*mstn*), atrophy, *gh*, and IGF systems as well as myogenic regulatory factors (MRFs). The IGF system, a major hormone axis regulating the cellular dynamics of muscle growth, directly stimulates cell proliferation, differentiation, and hypertrophy, and inhibits muscle atrophy. Such effects on muscle are mediated by the specific binding with IGF1 receptor (IGFR1) (23). In mice, previous studies showed that ablation of the IGF-1 receptor in skeletal muscle resulted in smaller myofibers (24). In rainbow trout, fasting and refeeding induced a coordinated regulation of IGF-I, IGFBP-5, and IGFBP-rP1 in muscle, and were suggested to be strongly involved in myogenesis resumption. Willoughby and Rosene (17) hypothesized that increased *mhc* gene expression induced by Cr supplementation is mediated by MRFs, which are transcription factors (*myod, myf5, mrf4*, and myogenin) that regulate myogenesis. In fact, *mrf4* level was increased after Cr intake in combination with resistance training. Increased *mrf4* and myogenin protein were further correlated to muscle CK mRNA expression (25). Safdar et al. (26) showed that short-term Cr supplementation for 10 days in young men increases the expression of numerous genes involved in osmotic regulation, glycogen synthesis and degradation, cytoskeletal remodeling, proliferation and differentiation of satellite cells, repairs and replication of DNA, RNA transcriptional control, and cell death. Furthermore, Young and Young (27) suggested that the beneficial effects of Cr supplementation in rat muscle mass and strength are due to an enhanced ability to train, rather than a direct effect on muscle. Hence, the potential anabolic effects of Cr might depend on the adjustment of workout intensity during training.

Although the majority of Cr research is focused in humans, its effect on other mammalian species meat quality has also been studied. Cr supplementation in pork diets prior to slaughter seems to affect the post-mortem muscle metabolism (pH decline in the muscle) and to improve the pork quality (28). The importance of the Cr system in fish still remains to be largely unknown, although, according to Borchel et al. (29), Cr metabolism differs between mammals and rainbow trout. It has been demonstrated that fish muscle has higher Cr content than that of mammals (30). McFarlane et al. (31) found that exogenous Cr supplementation (dietary or injected) did not alter rainbow trout muscle Cr levels, but during a fixed velocity sprint test, increased endurance was concomitantly observed with Cr intake. The short time frame of this study (7 days) associated with a too low dose to detect similar changes as seen in humans, given the lower metabolic rates of these poikilotherms, might explain the lack of Cr uptake in supplemented fish (31).

Relatively, less information is available on the Cr system of fish, and the effects of its dietary supplementation on muscle cellularity have never been evaluated before. The present study aims to contribute to a better understanding of the effects of dietary Cr supplementation levels on *S. aurata* juvenile's muscular growth. A comprehensive approach was undertaken based on the histological parameters (cellularity of the fast twitch muscle) and molecular biology techniques (relative expression of muscle-related genes).

## Materials and Methods

### Experimental Diets

A practical commercial-based diet, i.e., a control (CTRL), was formulated (49% protein and 23 kJ.g^−1^) to fulfill the known nutritional requirements of the gilthead seabream (Table 1). Three experimental diets were formulated by adding 2, 5, and 8% Cr monohydrate (Sigma, Ref. C3630) to the CTRL diet. All diets were manufactured by SPAROS (Olhão, Portugal). The main ingredients were pulverized (below 250 μm) in a micropulverizer hammer mill (Hosokawa Micron Ltd., United Kingdom) and mixed in a double-helix mixture (TGC Extrusion, France) to attain a basal mixture (no oils were added at this stage). All diets were extruded (pellet size 5.0 mm) by means of a pilot-scale twin-screw extruder CLEXTRAL BC45 (Clextral, France) with a screw diameter of 55.5 mm and temperature ranging 105°-110°C. Upon extrusion, all batches were dried in a convection oven (OP 750-EF, LTE Scientifics, United Kingdom) for 2 h at 60°C and left to cool at room temperature. The Cr was mixed with fish oil fraction according to each target concentration (2, 5, and 8%) and added under vacuum coating conditions in a Pegasus vacuum mixer (PG-10VCLAB, DINNISEN, The Netherlands) to the respective mixture.

**Table 1:** Ingredients and proximate composition of the control (CTRL) diet^*^.

**Ingredients**	**%**
Fishmeal LT^a^	10.00
Fishmeal 60^b^	10.00
Porcine blood meal	5.00
Soy protein concentrate^c^	10.00
Wheat gluten^d^	10.00
Corn gluten^e^	7.25
Rise protein concentrate	3.50
Soybean meal^f^	10.00
Rapeseed meal	4.00
Wheat meal	12.00
Fish oil^g^	14.50
Vit & Min Premix^h^	0.15
Soy lecithin^i^	2.00
Antioxidant	0.40
Dicalcium Phosphate^j^	0.50
L-Lysine^k^	0.50
DL-Methionine	0.20
**PROXIMATE COMPOSITION**	
Dry Matter (%)	95.39 ± 0.04
Crude protein (%DM)	49.28 ± 0.14
Lipid (%DM)	20.37 ± 0.31
Ash (%DM)	8.39 ± 0.06
Gross energy (kJ/g DM)	23.43 ± 0.07

a *Peruvian fishmeal LT: 71% crude protein, 11% crude fat, EXALMAR, Peru*.

b *Fish by-products meal: 540 g Kg^−1^ CP, 80 g kg^−1^ CF, COFACO, Portugal*.

c *Soycomil P: 65% CP, 0.7% CF, ADM, The Netherlands*.

d *VITEN: 85.7% CP, 1.3% CF, ROQUETTE, France*.

e *GLUTALYS: 61% CP, 8% CF, ROQUETTE, France*.

f *Solvent extracted dehulled soybean meal: 47% CP, 2.6% CF, SORGAL, Portugal*.

g *Henry Lamotte Oils GmbH, Germany*.

h *PVO40.01 SPAROS standard premix for marine fish, PREMIX Lda, Portugal*.

i *Yelkinol AC (65% phospholipids): 750 g Kg^−1^ CF,ADM, The Netherlands*.

j *Dicalcium phosphate: 18% phosphorus, 23% calcium, Fosfitalia, Italy*.

k *L-Lysine HCl 99%: Ajinomoto Eurolysine SAS, France*.

* *Experimental diets (Cr 2, 5, and 8%) were formulated by adding 2%, 5% and 8% Cr monohydrate (Sigma, Ref. C3630) to the CTRL diet*.

### Animal Growth Conditions

The current trial was conducted by trained scientists (following FELASA category C recommendations) and according to the European Economic Community animal experimentation guidelines on the protection of animals used for scientific purposes from the European directive 2010/63/UE at Ramalhete, CCMAR facilities (Centre of Marine Sciences of Algarve).

Triplicate groups of 24 gilthead seabream (initial body weight: 173 ± 2.4 g) were randomly distributed in 500 L tanks and were hand-fed *ad libitum* with each experimental diet twice a day (except Sundays) for 69 days. Sea water was supplied at 2 L/min (mean temperature 23.3°C ± 0.90; mean salinity 37 ± 0.39 ppm) in a flow through system with artificial aeration (mean dissolved oxygen above 5 mg.L^−1^). All physical and chemical water parameters were evaluated during the experiment to ensure the levels within the recommended limits for the species.

### Sampling

**Figure 1:**
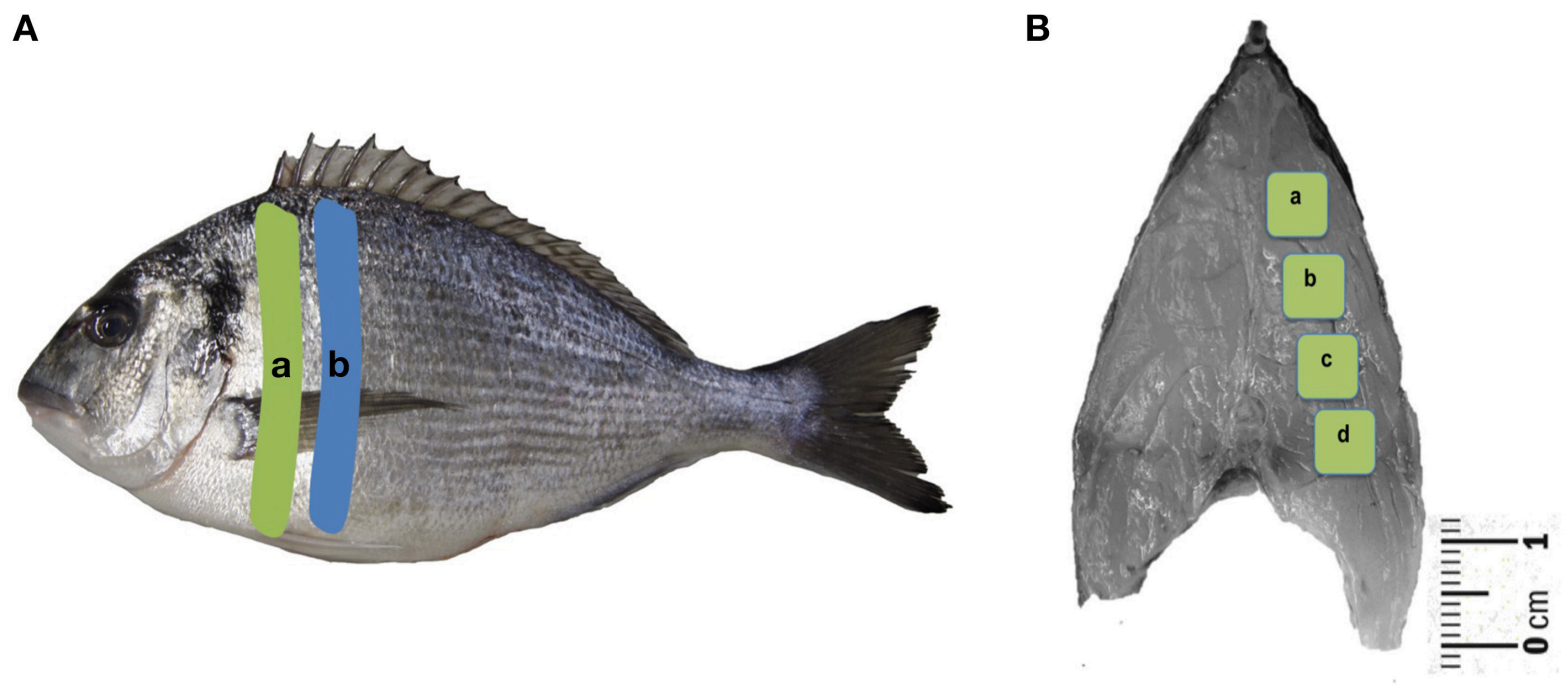
**(A)** Filet sampling area for histology parameters (a) and for molecular biology analysis (b) and **(B)** dorsal areas (a–d) selected for muscle cellularity evaluation.

At the end of the experimental trial, all fish were deeply anesthetized in an aqueous solution of MS-222 (Sigma, Switzerland) and individually weighted to calculate the daily growth index [DGI = 100 × (FBW^1/3^-IBW^1/3^)/trial duration (days)]. Six fish from dietary treatment were also measured for total standard length (cm) and sacrificed by decapitation under a cork board on ice. Their fins were cut and fish were softly scaled on both sides. A cross-sectional filet with skin (2–3 mm thick) was taken immediately before the dorsal fin position—filet A (Figure 1a). The dorsal area of each filet was then quickly photographed (with scale reference) and properly labeled, for later determination of the cross-sectional area. Four representative samples (a-c) of fast-twist muscle (0.5 × 0.5 cm) were collected from the right part of the filet (Figure 1B), immediately placed in a cryoprotective embedding medium—OCT (Thermo Scientific™ Shandon™ Cryomatrix™), and snap frozen in isopentane cooled by liquid nitrogen. Samples were then stored at −80°C for later morphometric evaluations.

A second cross-sectional filet (Figure 1A), filet B, was taken and 2–3 g of fast-twist muscle (right filet) was taken and stored in RNA*later*^TM^ solution (Sigma-Aldrich, USA) overnight at 4°C. The excess solution was then discarded and the samples were stored at −80°C for posterior molecular biology analysis.

### Analytic Methods

#### Morphometric Procedures

The morphometric study was done using an interactive image analysis system (Olympus Cell^*^Family), working with a live-image captured by CCD-video camera (ColorView Soft Imaging System, Olympus) and a light microscope (BX51, Olympus, Japan). Muscle total dorsal muscular area (DMA) (mm^2^) was computed by the software after demarcating the physical limits of the whole dorsal section without considering any red muscle area. These measurements were based on the photo taken at the sampling time.

Transversal fast-twist muscle sections from each block (a-d) were cut at 7 μm in a cryostat CM 1950 (Leica Microsystem GmbH, Wetzlar, Germany) and mounted on polysine adhesion slides. The sections were stained with haematoxylin–eosin (Merk, Whitehouse Station, NJ, USA) before placing a cover slip and left to dry. The relative number (density) of fast-twist muscle fibers per unit area N_A_(n°/mm^2^) was estimated as follows: N/area = *Σ* N(fibers)/*Σ* [a (sampled field)], where *Σ*N (fibers) is the total number of fibers counted over the sampled fields in the sections (a–d), and “a” is the total area of the fiber counting fields. The total number of fast-twist muscle fibers per dorsal cross-section (N) was estimated as follows: N (fibers) = N_A_ (muscle fibers) × DMA (muscle), where N_A_ is the number of fast-twist muscle fibers per unit area (mm^2^) and DMA the dorsal cross-sectional muscle area. From each fish, the physical limits of a minimum of 700 white muscle fibers (from the four blocks a-d) were circumscribed using a 20x objective to determine the mean fiber area [ā (μm^2^)]. The corresponding mean diameter was calculated assuming that all the fibers were circular.

#### RNA Extraction and cDNA Synthesis

White muscle samples were disrupted with a PureZol solution (Bio-Rad Laboratories) using Precellys® 24 lysis/homogenizer (Bertin Technologies, France). Total RNA was extracted using the Ilustra RNAspin Mini RNA isolation kit (GE Healthcare UK Limited), including an on-column DNAse I digesting step, according to the manufacturer's instructions. RNA quantification and quality were evaluated by absorbance at 260 and 280 nm using the Take3 Micro-Volume plate (Take3, Biotek, Germany) and the Gen 5 software (BioTek, USA), and the values were within the expected ratio of 1.8–2.2, indicating high RNA purity. RNA integrity was verified by the banding pattern of 28S:18S ribosomal RNA in 1% TAE (w/v) agarose gel electrophoresis stained with GelRed (Biotium, Hayward CA, USA).

For complementary deoxyribonucleic acid (cDNA) synthesis, 750 ng of total RNA was transcribed for all samples, with the iScript™ Reverse Transcription Supermix for real-time polymerase chain reaction (RT-qPCR) (Bio-Rad Laboratories) in a final volume of 20 μL following the manufacturer's instructions and stored at −80°C.

#### Real Time PCR Analysis

Primers used for qPCR had been previously published (Table 2) and were synthesized by STABVida (Portugal). The qPCR reactions were performed in iQ5 Real-Time PCR Detection System (Bio-Rad), using SsoFast EvaGreen Supermix (Bio-Rad Laboratories), and prepared to a final volume of 20 μl, with a final primers concentration of 300 nM, according to the manufacturer's instructions. Thermal cycling for these experiments occurred under the following conditions: initial step at 95°C for 30 s, followed by 40 cycles of denaturation at 95°C for 5 s, and plus annealing/extension (annealing temperatures in Table 2) for 10 s.

**Table 2:** List of specific primers used for real time PCR.

	**Primer Sequence 5^**′**^-3^**′**^**	**Annealing T.(°C)**	**Accession number**	**Reference**
**TARGET GENES**				
*mstn*	**F:** GTACGACGTGCTGGGAGACG	60	AF258448.1	(32)
	**R:** CGTACGATTCGATTCGCTTG			
*myod2*	**F:** CACTACAGCGGGGATTCAGAC	60	AF478568	(32)
	**R:** CGTTTGCTTCTCCTGGACTC			
*mrf4*	**F:** CATCCCACAGCTTTAAAGGCA	60	JN034421	(32)
	**R:** GAGGACGCCGAAGATTCACT			
*myogenin*	**F:** CAGAGGCTGCCCAAGGTCGAG	68	EF462191	(32)
	**R:** CAGGTGCTGCCCGAACTGGGCTCG			
*myf5*	**F:** TGTCTTATCGCCCAAAGTGTC	64	JN034420	(32)
	**R:** CTACGAGAGCAGGTGGAGAACT			
*myod1*	**F:** GTTTTGTTCCAGGCGGTCT	60	AF478569	(33)
	**R:** GCTGGTGTCGGTGGAGAT			
*mhc*	**F:** AGCAGATCAAGAGGAACAGCC	60	NM131404	(33)
	**R:** GACTCAGAAGCCTGGCGATT			
*capn1*	**F:** CCTACGAGATGAGGATGGCT	58	AM951595.1	(34)
	**R:** AGTTGTCAAAGTCGGCGGT			
*capn2*	**F:** ACCCACGCTCAGACGGCAAA	61	FM152855.1	(34)
	**R:** CGTTCCCGCTGTCATCCATCA			
*capns1a*	**F:** CGCAGATACAGCGATGAAAA	56	AM962179.1	(34)
	**R:** GTTTTGAAGGAACGGCACAT			
*capns1b*	**F:** ATGGACAGCGACAGCACA	56	ERP000874	(34)
	**R:** AGAGGTATTTGAACTCGTGGAAG			
*capn3*	**F:** AGAGGGTTTCAGCCTTGAGA	56	FG262721.1	(34)
	**R:** CGCTTTGATCTTTCTCCACA			
*igfr-1a*	**F:** TCAACGACAAGTACGACTACCGCTGCT	60	KJ591052	
	**R:** CACACTTTCTGGCACTGGTTGGAGGTC			
*igfr-2*	**F:** ACATTCGGGCAGCACTCCTAAGAT	60	KM522776	
	**R:** CCAGTTCACCTCGTAGCGACAGTT			
*ghra*	**F:** ACCTGTCAGCCACCACATGA	60	AF438176	
	**R:** TCGTGCAGATCTGGGTCGTA			
**REFERENCE GENES**				
***β-actin***	**F:** TCCTGCGGAATCCATGAGA	60	X89920	(34)
	**R:** GACGTCGCACTTCATGATGCT			
***rpl27α***	**F:** AAGAGGAACACAACTCACTGCCCCA	68	–	(35)
	**R:** GCTTGCCTTTGCCCAGAACTTTGTAG			
***18S***	**F:** CGAGCAATAACAGGTCTGTG	60	–	(36)
	**R:** GGGCATGGACTTAATCAA			

*For each gene, the annealing temperature and the gene bank accession number, whenever available, are indicated*.

Then the melting curve analysis was performed to verify the amplicon purity and size, with a dissociation protocol from 65° to 95°C followed by gel electrophoresis. Five-point standard curves constructed with 5-fold serial dilutions of pooled cDNA were used for qPCR efficiency calculation. All samples were performed in duplicated and always included a negative control to confirm the absence of contamination. To evaluate the relative transcript levels, the 2^−ΔΔCT^ method was used with β*-actin* and *rpl27*α as the best housekeeping genes out of three, estimated by geNorm® software, to provide the most reliable normalization. The PCR efficiency for target genes ranged from 85 to 110%.

#### Statistical Analysis

Statistic evaluation of the data was accomplished by one-way analysis of variance (ANOVA). All data were checked for normality and homogeneity of variance, by using the Shapiro-Wilk and the Levene test, respectively. Data transformation [log(x) and arcsin(x)] was applied when homogeneity and normality of the variables were not achieved. A non-parametric test (Kruskal-Wallis H-test) was performed, if these assumptions where still not achieved. A pair-wise Mann–Whitney U-test was used for *post-hoc* multiple comparisons. Where significant main effects were identified by ANOVA, individual means were compared using *Tukey HSD* multiple comparison test. A significance of *p* < 0.05 was applied to all statistical tests. A Spearmen's rank correlation coefficient (ρ) test was applied to all variables. Correlation was considered significant at the bilateral levels of 0.05 (*) or 0.01 (**). All tests were run with IBM SPSS statistics software (SPSS ver.22.0; Chicago, USA).

The evaluation of expression stability for the three reference genes was performed using the statistical application geNorm® (https://genorm.cmgg.be).

## Results

### Muscle Growth

During the experimental period, no mortalities were registered and all fish reached the commercial size (>250 g). There was a general trend for Cr-fed fish to be larger and longer than those fed the CTRL, but without differing significantly (Table 3). Condition factor (K), used as an index of the productivity in fish growth, ranged from 2.3 to 2.4 with no significant differences between treatments, nonetheless was higher in fish fed the highest Cr inclusion. No significant differences in daily growth index (DGI) were registered among the dietary treatments.

**Table 3:** Growth performance and muscle cellularity of gilthead seabream juveniles fed CTRL, Cr 2, 5, and 8% diets^*^.

	**Diets**			
	**CTRL**	**Cr 2%**	**Cr 5%**	**Cr 8%**
Final Weight (g)	272.14 ± 18.92	274.98 ± 17.36	291.29 ± 23.60	288.32 ± 29.32
Length (cm)	22.75 ± 0.90	22.86 ± 0.39	22.96 ± 0.73	22.92 ± 0.45
Condition factor (K)	2.31 ± 0.13	2.26 ± 0.27	2.28 ± 0.31	2.43 ± 0.24
DMA (mm^2^)	771.83 ± 46.99^b^	798.44 ± 71.69^b^	933.04 ± 22.16^a^	899.51 ± 82.98^a^
Fiber Density (N/mm^2^)	170.47 ± 12.94	166 ± 18.80	166.55 ± 21.14	149.18 ± 12.32
Dorsal total fiber number x1000	131.40 ± 10.57	132.98 ± 22.66	150.75 ± 16.61	134.37 ± 16.55
Diameter of fibers (μm)	69.06 ± 2.38	69.59 ± 4.62	70.75 ± 2.67	73.71 ± 3.70
Fibers ≤20μm (%)	1.49 ± 1.12	1.99 ± 1.52	1.91 ± 1.68	1.65 ± 1.29
Fibers ≥120μm (%)	8.97 ± 1.74	9.19 ± 3.36	9.35 ± 1.81	12.19 ± 3.52

* *Values represent the mean ± standard deviation (n = 6). Mean values within a row with different letters (a, b) represent significant differences between diets (P < 0.05). DMA, dorsal cross-sectional muscle area*.

**Figure 2:**
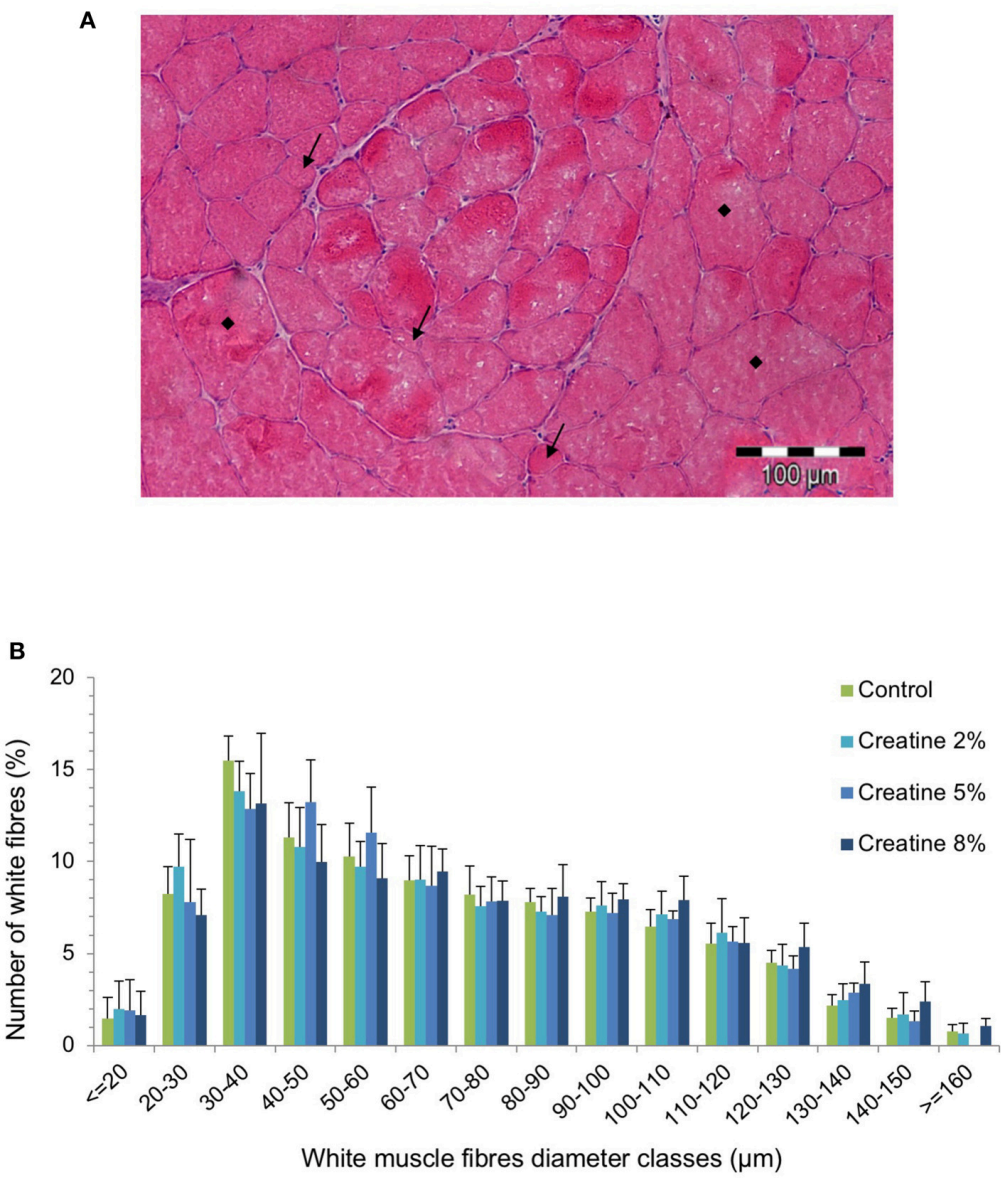
Cross section of skeletal white muscle in a juvenile gilthead seabream fed 5% Creatine diet, showing newly (i.e., small [arrow]) recruited muscle fibers between older (i.e., large ■) muscle fibers **(A)**, and white muscle fiber diameter classes of juveniles fed juveniles fed the experimental diets for 69 days (*n* > 700 fibers) **(B)**. Error bars indicate the standard error of the mean for each treatment (*n* = 6).

The dorsal muscular area (DMA) of fish fed with Cr 5 and Cr 8% was significantly larger than that of fish fed with CTRL and Cr 2% diets (*P* < 0.05; Table 3). Dorsal total fiber number was highest in fish fed with 5% Cr, but no significant differences could be perceived among dietary treatments. The mean diameter of fast-twist fibers had a tendency to increase with Cr supplementation, whereas fiber density showed an inverse trend (Table 3). In addition, the distribution of skeletal fast-twist fiber diameters showed no significant diet-induced differences (Figure 2B). Muscle fiber diameter ranged from <20 μm to a maximum of 160 μm (Figures 2A,B). The groups supplemented with creatine systematically had a higher relative number of both small-sized (≤20 μm) and large-sized fibers (≥120 μm) (Table 3).

### Relative Expression of Target Genes

**Figure 3:**
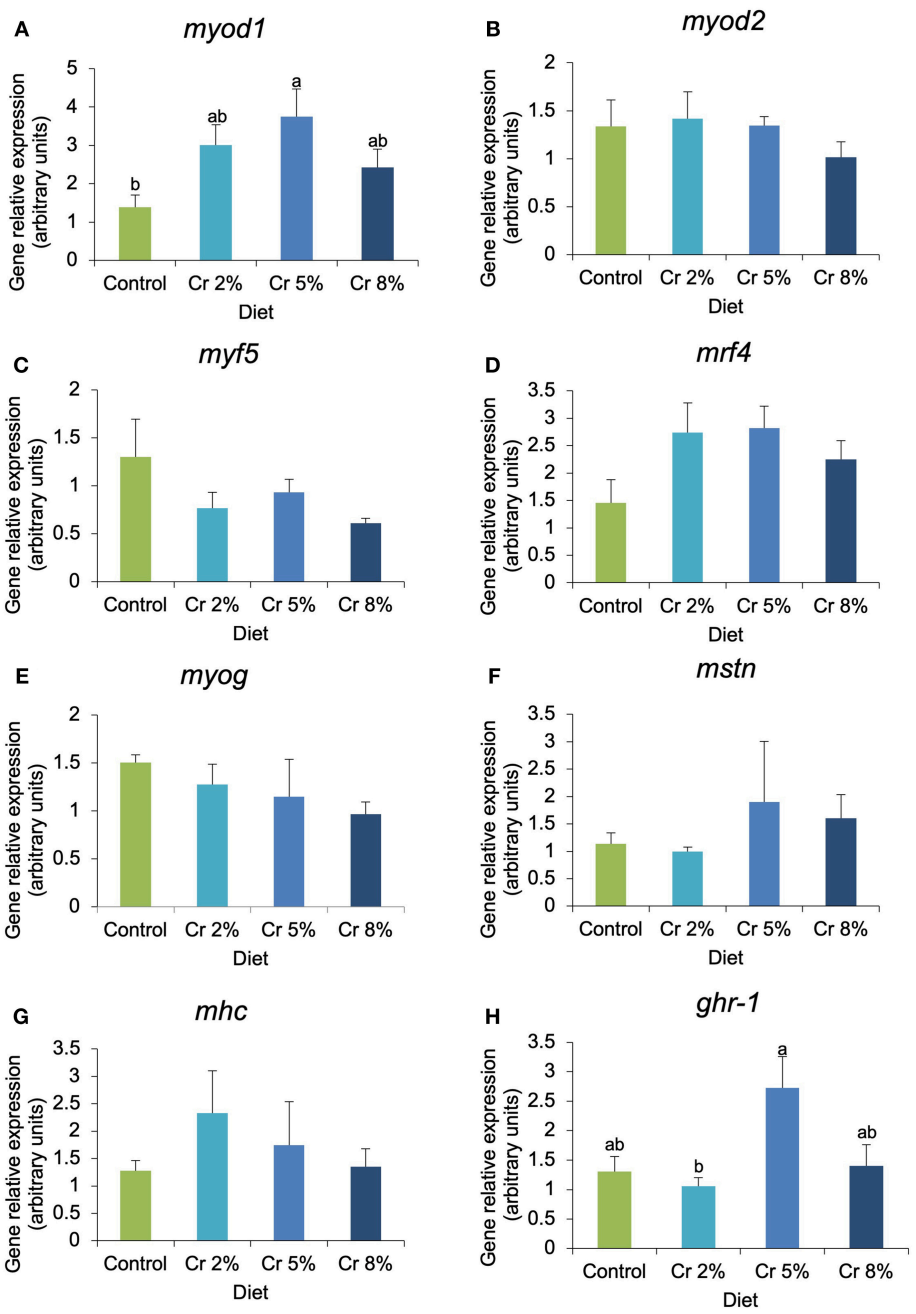
**(A–H)** Relative expression of myogenic genes and markers of muscle structure and function of gilthead seabream juveniles fed the control and the experimental diets (2, 5, and 8% creatine). Different letters indicate significant differences between groups. *P* < 0.05. Error bars indicate the standard error for each treatment (*n* = 6).

**Figure 4:**
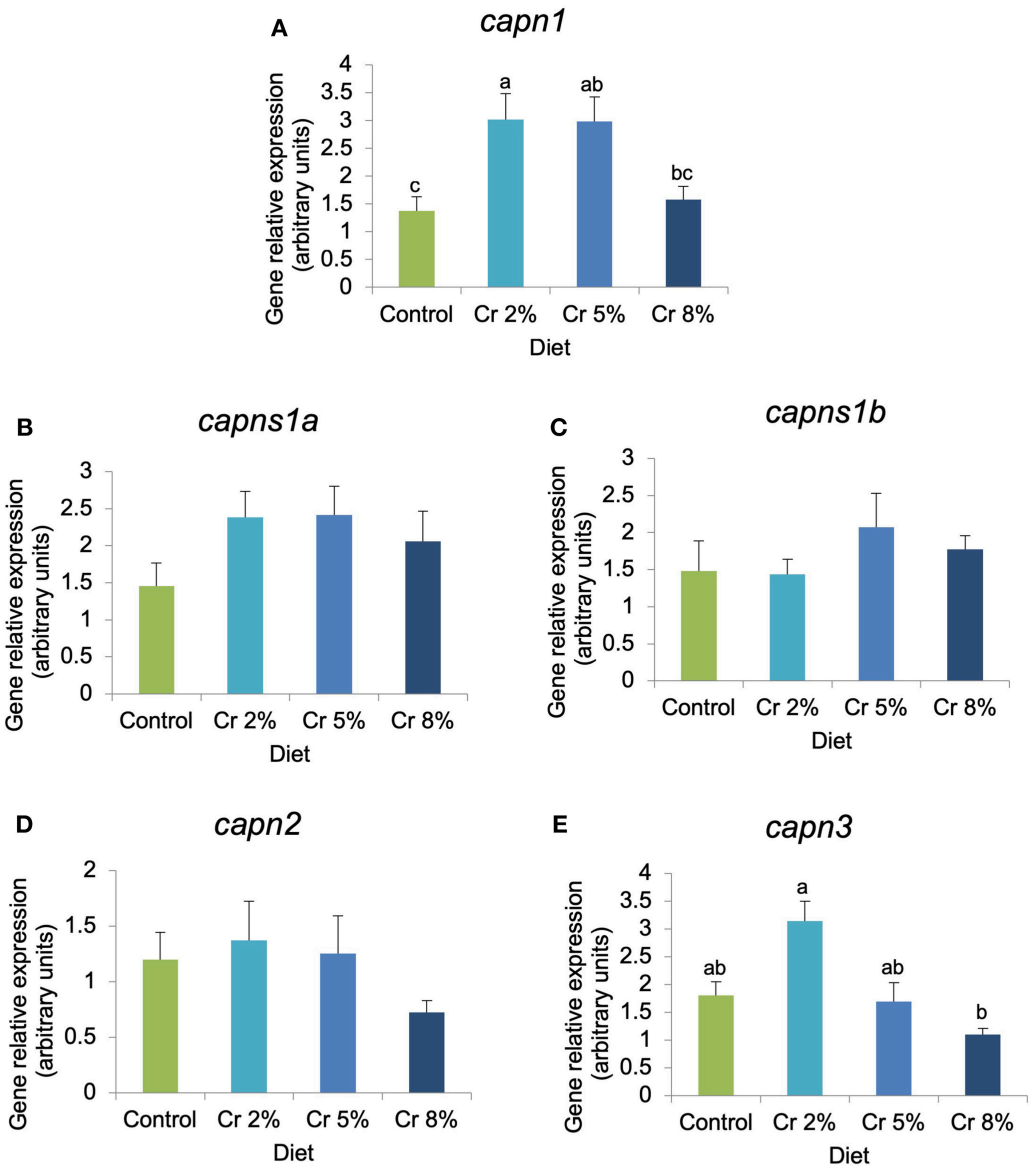
**(A–E)** Relative expression of genes involved in proteolysis in gilthead seabream juveniles fed the control and the experimental diets (2, 5, and 8% creatine). Different letters indicate significant differences between groups. *P* < 0.05. Error bars indicate the standard error for each treatment (*n* = 6).

In fast-twitch muscle, the expression of *myod1, capn1*, and *capn3* was significantly affected by the dietary treatments, whereas other myogenic genes (*myod2, myf5, mrf4*, and *myog*) and biomarkers of muscle structure, function, and growth (*igfr-1a, igfr-2, mhc, mstn, capns1a, capns1a*, and *capn2*) were not significantly changed (Figures 3, 4). In fish supplemented with 5% Cr, the relative expression of *myod1* increased almost four times compared with those fed with the CTRL diet (*P* = 0.045; Figure 3A). The *mrf4* had the very same trend of *myod1* but changes were not significant. The relative expression of *ghr-*1 increased almost three times in fish fed with 5% Cr compared with those fed with 2% Cr (*P* = 0.041; Figure 3H) but did not differ significantly from other treatments. The relative expression of both *myf5* and *myog* tended to decrease with increasing Cr supplementation but without statistical significance. In addition, the expression of calpain 1 (*capn1*) increased significantly in fish fed with Cr 2 and Cr 5% (*P* = 0.005; Figure 4A). On the other hand, fish fed with 8% Cr showed a similar *capn1* expression to those fed with the CTRL diet. The relative expression of *capn3* was highest in fish fed with 2% Cr supplementation but did not differ significantly from those fed with the CTRL or Cr 5%. Fish fed with Cr 8% diet had the lowest *capn3* expression.

To better understand the possible relationship between the relative expression of muscle-related genes and the muscle cellularity, a Spearman rank order correlation was performed with all parameters (Table 4). The expression of the majority of the genes was not significantly correlated with muscle phenotype. However, a positive correlation was found between *mstn* and fiber diameter (*P* = 0.664), whereas *myog* expression levels were negatively correlated with DMA (*P* = −0.622). Interestingly, the expression of several genes implicated in myogenesis was significantly correlated with the expression of genes from the calpain family. Both *myod* paralogs in muscle (*myod1* and *myod2*) were positively correlated with almost all the genes from the calpain family analyzed herein (Table 4). The *myod1* gene had a positive and significant correlation with *capn1, capns1a*, and *capn3* expression. Similarly, *myod2* showed a strong positive correlation with *capn1* (ρ = 0.727), *capns1a* (ρ = 0.643), *capn2* (ρ = 0.594), and *capn3* (ρ = 0.762) expressions. *Myf5* was also significantly correlated with *capn2* (ρ = 0.769) and *mrf4* with *capn1* (ρ = 0.790) expression (Table 4).

**Table 4:** Correlations between gene expression and muscle growth parameters (DMA and fiber diameter) in gilthead seabream juveniles.

	**DMA**	**Fiber diameter**	***capn1***	***capns1a***	***capn2***	***capn3***
*myod1*	NS	NS	ρ = 0.804^**^	ρ = 0.650^*^	NS	ρ = 0.580^*^
*myod2*	NS	NS	ρ = 0.727^**^	ρ = 0.643^**^	ρ = 0.594^*^	ρ = 0.762^**^
*myf5*	NS	NS	NS	NS	ρ = 0.769^**^	NS
*mrf4*	NS	NS	ρ = 0.790^**^	NS	NS	NS
*mstn*	NS	ρ = 0.664^*^	NS	NS	NS	NS
*myog*	ρ = −0.622^*^	NS	NS	NS	NS	NS

NS, not significant. Significance levels set at P < 0.05 (*) and P < 0.01 (**).

*DMA, dorsal cross-sectional muscle area*.

## Discussion

Cr supplementation has been used for many years by athletes to promote body mass growth and to improve their training resistance. A relatively large number of scientific studies have associated with the increased lean body mass to Cr supplementation combined with strength training (15, 16, 37); however, it is still not very clear whether the Cr supplementation *per se* is enough to promote such effects (38). Studies concerning the effect of Cr, although widely disseminated with regard to humans and mammal species, are extremely scarce in teleost fish. This study has been conducted to evaluate the potential of Cr supplementation to improve gilthead seabream muscle growth and the possibility of tailoring filet quality to fulfill the consumers' expectations.

The present results show that Cr supplementation does not seem to be very effective in promoting body mass increase in gilthead seabream, as fish final weight and DGI were not significantly improved after 69 days of feeding. Similarly, a short-term (7 days) dietary Cr supplementation did not significantly affect the specific growth rate (% body weight change d^−1^) in juvenile rainbow trout (31). Nevertheless, the present study shows that the supplementation of Cr up to 5% in diets for gilthead seabream resulted in a significant increase of fish DMA. This was associated with a concomitant increase in muscle fiber diameter (muscle hypertrophy), mainly due to increased number of large-sized fibers (≥120 μm) and higher total number of fiber in those fish. It is well-known that the skeletal muscle cellularity (i.e., the number, diameter, and density of fibers) is the main determinant of muscle texture both in raw and cooked filet, and is directly related with fish growth potential (4). In gilthead seabream, previous studies showed that flesh firmness was positively correlated with both the fiber density and the number of small fiber, and negatively correlated with skeletal muscle diameter (9). The present results suggest that in gilthead seabream, the dietary Cr supplementation *per se* significantly increased the DMA but was not enough to promote significant effects on the muscle fiber cellularity after a 69-day feeding period. Although fish muscle Cr and PCr levels are less susceptible of manipulation than human muscle stores, either by dietary supplementation or injection (31), a longer feeding period or the conjugation with resistance training might further result in a significant stimulus to growth but could also have a negative impact on flesh texture parameters due to increased muscle fiber diameters. Further studies are required to clarify such potential effects. In spite of the differences regarding the metabolism of Cr between mammals and fish (29), it has been demonstrated that Cr supplementation associated with exercise resulted in muscle thickness improvement in young athletes (39). However, the controversy regarding this subject persists; for instance, in a work with rats conducted by Aguiar et al. (40), dietary Cr supplementation did not significantly affect fiber hypertrophy neither when used alone nor when the rats were subjected to resistance training.

The growth potential of fish is intrinsically dependent on post-natal hyperplasia and hypertrophy dynamics in muscle tissue, which is controlled by signaling pathways involving the growth hormone (*gh*)/insulin-like growth factor (*igf*) system. *Gh* and *igfs* stimulate somatic growth through binding their corresponding receptors (*gh* and *igfrs*, respectively) that are widely distributed among different tissues, including muscle, and are influenced by the nutritional status of fish (22, 41). Previous studies reported that in humans, high Cr supplementation enhanced GH secretion, mimicking the response of a strong exercise, which might result in acute body weight and strength gain probably due to the indirect anabolic property of Cr (41). In addition, Cr supplementation at rest increased the muscular expression of the insulin-like growth factors that are extremely important growth-promoting agents (20). In fish, the impact of dietary supplementation of Cr in the GH/IGF system has never been reported before. In the present study, an up-regulation of *ghra* was observed in fish fed with 5% Cr diet (the relative expression increased by 110%), the gene that has prominent role in the systemic growth-promoting action of Gh (42, 43), whereas both *igfr-1a* and *igfr-2* remained unaffected. Vélez et al. (44) also reported an up-regulation of *ghra* in the muscle of gilthead sea fingerlings as the effect of rBGH treatment, suggesting that the GH anabolic effects may be induced in this tissue directly through the activation of this receptor.

Previous studies showed that a higher number of small-sized fibers are associated with higher growth potential (4, 45), which in turn depend on the proliferation and differentiation of the myogenic progenitor cells (MPCs, equivalent to mammalian satellite cells) that are responsible for controlling the expression of muscle-related genes. Myogenic activity is regulated by the differential expression of MRFs, which are transcription factors involved in the proliferation and differentiation of MPCs (46). The *myod* (myoblast determination factor) and *myf5* are primary MRFS involved in the specification and proliferation of myoblasts to form the MPC population. These cells, after activation and proliferation, will enter the differentiation process that will result in myotube formation and enlargement, involving the expression of the secondary MRFs *(myog and mrf4)* (46). There is a lack of surveys dedicated to the effect of dietary Cr on vertebrate's myogenic program. In the present study, the *myod1* relative expression significantly increased concomitantly with Cr dietary supplementation. The highest expression was observed in fish fed 5% Cr, suggesting that an increase in myoblast recruitment was occurring. During the muscle differentiation process in adult fish, such new cells fuse to form additional fibers or are absorbed by the existing fibers as they expand in diameter (hypertrophic growth) (47).

The currently observed up-regulation of *myod1* in gilthead seabream fed diets supplemented with 2–5% Cr was paralleled with a significant increase in DMA (*myod1* relative expression increased 167%, whereas DMA increased 21 % in fish fed with 5% inclusion of Cr in relation to the control diet). This was probably due to the concomitant increase in total number and size of muscle fibers. Moreover, *myod2* transcripts levels have not only showed lower levels compared with those of *myod1* but also were not significantly affected by dietary Cr. Similarly, in gilthead seabream, a differential expression of *myod1* and *myod2* was observed in amino acid-deficient media (48), also suggesting a differential nutritional regulation of the two *myod* paralogs. According to Tan and Du (49), the two non-allelic *myoD* genes are functional in seabream adult skeletal muscles and their expression is regulated differently: MyoD1 is expressed in both slow and fast muscles, whereas MyoD2 is specifically expressed in fast muscles (49). Campos et al. (50) have previously shown that in Senegalese sole larvae, *myod1* was correlated with fiber diameter, but not *myod2*. Moreover, in the present study, only *mstn* evidenced a negative correlation with fiber diameter. Overall, this indicates that the observed nutritional regulation can vary depending on the fish species and the stage of myogenesis of the muscle under study. Aguiar et al. (51) found a strong correlation between the muscle fiber CSA and the expression of *myod* in an experiment of resistance training in rats. The authors argued that this factor is more involved in the control of muscle mass than in fiber-type transitions (51). Accordingly, Siqin et al. recently explored the relationships among muscle fiber-type composition, diameter, and MRFs expression in different skeletal muscles, they also suggested that MRFs expression patterns were relatively stable with the changes in fiber-type composition and increases in fiber size resulting from mutually interacting processes during muscle development (52). Furthermore, Deldicque et al. (53) identified a major signaling cascade by which Cr promotes the differentiation program of C_2_C_12_ cells, via p38 MAPK and ERK1/2 pathway, which may increase the expression of transcription factors (i.e., *myod* and *mef2*) capable of regulating the activation and differentiation of satellite cells. Studies in humans reported an increase in the expression of both myogenin and *mrf4* levels after Cr supplementation in conjunction with resistance training, which were strongly correlated with muscle Cr kinase mRNA expression (25), but other studies did not observe any significant changes in myogenin expression (54). In our study, *mrf4* expression tended to increase with Cr supplementation (43% increase in fish fed with Cr 5% compared with those fed with the CTRL diet), but *myog* expression even showed a downward trend in relative expression from Cr 2 to Cr 8%, which could foresee a decrease in fiber differentiation. Nevertheless, myostatin expression was positively correlated with muscle fiber diameter, although no clear trend could be perceived in its expression level in fish fed with increasing Cr levels. Both myogenin and myostatin are known to control myoblast differentiation and fusion that lead to the formation of myofibrils in several species (46). However, in Senegalese sole fed with different dietary, lipid diets *mstn* was negatively correlated with the percentage of large-sized fibers and with fish DMA (55). Data from previous works in aged mice reported a similar behavior and myostatin inhibitors having significant positive effects on muscle fiber size and mass (56, 57). Although recognized for repressing skeletal muscle growth through inhibiting both muscle cell hypertrophy and hyperplasia, in fish, recent studies suggested that *mstn1* seems to inhibit muscle cell proliferation, but not its differentiation (58). Thus, further research is needed to better clarify the effects of dietary Cr in *mstn*-associated muscular behavior.

The expression of several genes implicated in myogenesis was significantly correlated with the expression of genes from the calpain family. Calpains are a group of non-lysossomal Ca^2+^-dependent cysteine proteases involved in cell cycle progression, myoblasts fusion, muscle protein turnover and growth, cell mobility, and cell degradation (59). Although in fish the role of calpains remains controversial, these proteases are generally associated with flesh tenderization and with the post-mortem changes occurring in muscle (60). They act in synergy with cathepsins to contribute to a rapid proteolysis of muscle proteins and associated flesh softening during post-mortem storage of meat. *Capn1* and *capn2* regulate physiological processes like myoblast fusion, and *capn3* is known to play an important role in skeletal muscle homeostasis and protein turnover (35). Previous studies with gilthead seabream showed that the expressions of *capn1* and *capns1a* were inversely correlated with muscle texture, suggesting that they may serve as potential genetic markers of flesh quality (34). In salmonids, calpain activity also influences the filet quality but did not seem to substantially function in active muscle turnover (61). In cattle and sheep, a strong correlation has been observed between *capn3* expression levels and meat tenderness (shear force measurements), but no direct evidence could link *capn3* levels with fish (34) or porcine muscle texture (62–64).

In the present study, the relative expression of both *capn1* and *capn2* showed an overall increase with Cr supplementation up to 5%, and the expression of calpain 1 (*capn1*) increased significantly in fish fed with Cr 2 and Cr 5% in comparison with the CTRL group. Moreover, the *capn1* gene not only had a positive and significant correlation with *myod1* but also with *mrf4*, suggesting an important role in myoblast proliferation and fusion in response to Cr supplementation. A strong positive correlation among *capn3* and both *myod1* and *myod2* was also observed in the fast skeletal muscle of gilthead seabream juveniles fed with Cr-supplemented diets. In fish, information regarding the function of calpains on myogenesis is extremely scarce and its involvement in the regulation of MRFs remains largely unknown. However, in gilthead seabream, calpains were shown to be very important during the proliferation phase of early myogenesis, decreasing progressively with development (65). This suggests an anabolic aspect of calpains mainly involved in disassemble of sarcometric structure during muscle remodeling and cell fusion. This is supported by the present findings where a concomitant upregulation of *capn1, capn2*, and *myod* was observed in fish fed with Cr up to 5% resulting in increased myoblast proliferation and fiber hypertrophy. Notwithstanding, previous studies using μ-calpain (*capn1*) knockout mice reported an increase in size and number of fast-twitch glycolytic muscle fibers, indicating that mice with *capn1* suppressed exhibit an increased capacity to accumulate and maintain protein (i.e., proteins associated with muscle regeneration) in their skeletal muscle, and a decrease in *myod* expression, suggesting less muscle regeneration (66). Studies using C_2_C_12_ cells further demonstrated that *capn3* is involved in the myogenic differentiation process, affecting the establishment of the reserve cells pool by decreasing the transcriptional activity of the myod via proteolysis without affecting the other MRFs (67). However, it was shown unlikely that myod function within myotubes was affected due to the presence of high levels of myod. In fish, the proliferation of MPC continues largely after the juvenile stage contrarily to what is observed in higher vertebrates where hyperplasia stops after birth (33). It is, hence, probable that distinct processes may be involved in the regulation of the satellite cell compartment among species. In juvenile seabream, the activity of the myod or the capn3 levels was not evaluated, but the increased expression of *myod1* in fish fed with Cr suggests an activation and differentiation of cells that resulted in increased number and size of muscle fibers. Moreover, the upregulation of *capn3* might have increased muscle proteolysis in Cr-treated gilthead seabream juveniles, but the observed increase in dorsal muscle area also suggests a concomitant increase in protein synthesis probably resulting from the upregulation of *myod1*. Further studies are still required to fully understand the proteolytic system in fish and its implication on the myogenic program.

## Conclusions

From this study, we can conclude that the dietary Cr supplementation in gilthead seabream juveniles resulted in a significant increase in fish DMA. Dietary Cr *per se* significantly affected the expression of some genes related with myogenesis (*myod1*) and others involved in muscle texture and proteolysis (*capn1*), contributing to their upregulation in fish fed up to 5% Cr. Nevertheless, this modulation of gene expression was not enough to produce a significant alteration in muscle phenotype under the tested conditions because a non-significant increase in muscle fiber diameter and higher total number of fiber was observed, but still resulted in increased DMA. Additional studies may be required in order to better clarify the effect of dietary Cr supplementation in fish, possibly in conjunction with the induced resistance training. Moreover, supplementation during teleost's early life stages, where muscle growth is more pronounced (nutritional programming), and evaluation of filet yield and textural properties in commercial-sized fish need further research.

## Author Contributions

LV and PR conceived and designed the study. LR-P, GL, VS, LC, and DS performed all laboratorial work and collected data. LR-P and GL drafted the manuscript. All authors contributed to the interpretation and discussion of the data. The final version of the manuscript was approved by all the authors.

### Conflict of Interest Statement

The authors declare that the research was conducted in the absence of any commercial or financial relationships that could be construed as a potential conflict of interest.
